# Structure-based assessment and network analysis of targeting 14-3-3 proteins in prostate cancer

**DOI:** 10.1186/s12943-018-0905-y

**Published:** 2018-10-31

**Authors:** Alex Root, Azadeh Beizaei, H. Alexander Ebhardt

**Affiliations:** 1 Molecular Biology Program, Memorial Sloan Kettering Cancer Center, New York, NY USA; 2 Systems Biology Ireland, University College Dublin, Belfield, Dublin 4, Ireland

**Keywords:** BV02, Structure-based drug design, MM-GBSA, Docetaxel, 14-3-3 protein family, YWHAZ

## Abstract

Developing combination therapy for castrate-resistant prostate cancer (CRPC) may require exploiting new drug targets outside androgen receptor and PI3K / AKT / mTOR signal transduction pathways implicated in prostate cancer (PCa) progression. One such possible new target is YWHAZ of the 14-3-3 protein family as this gene has prognostic significance for metastatic CRPC patients. However, there are no small molecules targeting YWHAZ commercially available. Hence, we explored whether the small molecule BV02 targeting another 14-3-3 protein family member SFN also binds to YWHAZ. Using advanced docking algorithms we find that BV02 docks many other 14-3-3 family members. In addition, the amphipathic groove where drug binding occurs also has a high binding affinity for other drugs used to treat PCa such as docetaxel. The proteome of metastatic PCa models (LNCaP clone FGC and PC-3) was perturbed as a result of BV02 treatment. Through data integration of three proteomics data sets we found that BV02 modulates numerous protein-protein interactions involving 14-3-3 proteins in our PCa models.

**Electronic supplementary material:**

The online version of this article (10.1186/s12943-018-0905-y) contains supplementary material, which is available to authorized users.

Metastatic castrate-resistant prostate cancer (mCRPC) affects approximately 1 in 30 men in the United States and there is an unmet need for effective therapy. Many mCRPC patients receive the chemotherapy drug docetaxel which prevents microtubule depolymerization thus arresting cell division. Advances in drug design and development lead to new compounds targeting the androgen receptor (AR) as a treatment option for mCRPC [1]. Despite these advances, 29,430 men will die of prostate cancer (PCa) in the U.S.A in 2018 [2]. A simulation study based on survival data of 2353 PCa patients concluded that a two-drug combination therapy is unlikely to cure mCRPC patients [3]. Hence, searching for higher-order drug combinations targeting pathways in addition to PI3K/AKT/mTOR is warranted. One source for drug targets is recurrently amplified genomic regions. In approximately 30% of patients with metastatic PCa regions of chromosome 8q are amplified which includes genes like MYC, NCOA2, and YWHAZ. The latter protein was found to be upregulated as a function of pharmacological intervention [4]. Taken together, these studies suggest YWHAZ is a potential drug target for PCa therapy. Binding a large number of proteins makes the 14-3-3 family an attractive tool for modulating protein activity, and thereby controlling multiple signal transduction pathways suggesting its role as universal regulators [5].

A nonpeptidic small molecule inhibitor of SFN termed BV02 induces apoptosis in chronic myeloid leukaemia models [6]. Subsequent NMR studies determined that BV02 is a labile compound in aqueous solution and the phthalimide derivative of BV02 termed BV02_9 serves as bioactive molecule [7]. Given that YWHAZ has close homology with SFN we hypothesized that BV02 also inhibits YWHAZ.

## Results and discussion

### Assessment of binding affinities of multiple compounds with 14-3-3 family members using molecular mechanics

**Fig. 1:**
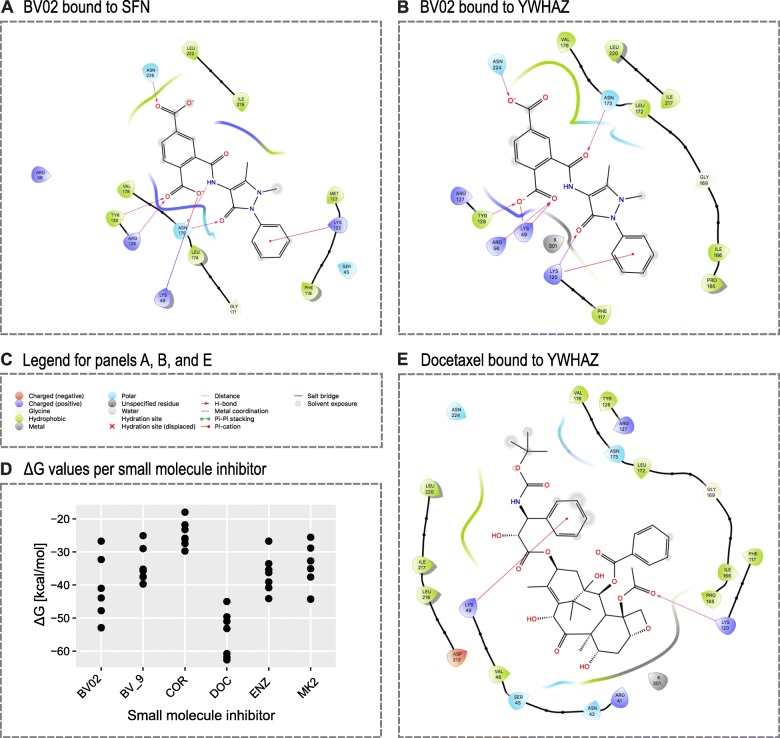
Results of docking studies. In color on the outside are amino acid residues while small molecules are represented with their chemical structure. H-bonds are represented as solid purple lines while pi-cation interactions are red. **a** Ligand interaction diagram between BV02 and SFN coordinated by H-bonds and pi-cation interaction. Several residues important for the protein-drug interaction include Lys-49, Arg-129, and Tyr-130. **b** Ligand interaction diagram between BV02 and YWHAZ involves different residues compared to SFN, e.g. Lys-49. **c** Figure legend for panels A, B, and E. **d** Free energy of binding (ΔG) for all seven 14–3-3 protein family member plotted for each small molecule inhibitors (BV02; BV02_9; COR: corannulene; DOC: docetaxel; ENZ: enzalutamide; MK2: MK2206). **e** Although docetaxel is only coordinated by a H-bond and a pi-cation interaction, the molecule fits squarely into the amphipathic binding groove resulting in very low ΔG values

To confirm our hypothesis, we used structure-based analysis to assess the binding affinity of BV02 to YWHAZ. To compare the docking of BV02 and other small molecules between all seven 14-3-3 protein family members we used the Molecular Mechanics Generalized-Born Solvent Accessibility method, implemented within the Schrodinger Software Suite. The resulting free energy values (ΔG) allow for direct comparison within our seven protein – six small molecule matrix. In case of BV02 binding to all seven 14-3-3 protein family members with examples of docking shown in Fig. 1a and b. Differential ΔG were observed between protein family members with ΔG values ranging from − 26.7 kcal/mol in case of YWHAB to − 53.0 kcal/mol for YWHAE (Fig. 1d, Table 1). The target protein SFN has a binding affinity of − 47.8 kcal/mol suggesting that BV02 is a promiscuous binder. The bioactive form BV02_9 has a smaller dynamic range for ΔG compared to BV02 with values ranging from − 25.1 kcal/mol (YWHAB) to − 39.7 kcal/mol (YWHAE) suggesting less specificity towards anyone 14-3-3 protein family member.

**Table 1:** Docking scores and binding free energy estimation with all 14–3-3 family members

Protein	Drug	MMGBSA ΔG Bind
SFN	Docetaxel	−50.995
SFN	BV02	−47.767
SFN	BV02_9	−37.437
SFN	Enzalutamide	−35.406
SFN	Corannulene	−27.451
SFN	MK2206	−25.558
YHWAQ	Docetaxel	−49.609
YHWAQ	MK2206	−44.319
YHWAQ	BV02	−41.003
YHWAQ	Enzalutamide	−36.277
YHWAQ	BV02_9	−28.979
YHWAQ	Corannulene	−26.181
YWHAB	Docetaxel	−44.997
YWHAB	MK2206	−35.065
YWHAB	Enzalutamide	−33.543
YWHAB	BV02	−26.734
YWHAB	BV02_9	−25.072
YWHAB	Corannulene	−21.792
YWHAE	Docetaxel	−60.766
YWHAE	BV02	−52.966
YWHAE	MK2206	−44.228
YWHAE	Enzalutamide	−44.112
YWHAE	BV02_9	−39.695
YWHAE	Corannulene	−17.976
YWHAG	Docetaxel	−61.867
YWHAG	Enzalutamide	−40.822
YWHAG	MK2206	−37.603
YWHAG	BV02_9	−35.213
YWHAG	BV02	−32.296
YWHAG	Corannulene	−25.861
YWHAH	Docetaxel	−53.17
YWHAH	BV02	−52.878
YWHAH	BV02_9	−35.691
YWHAH	Corannulene	−29.735
YWHAH	MK2206	−28.84
YWHAH	Enzalutamide	−26.734
YWHAZ	Docetaxel	−62.634
YWHAZ	BV02	−43.919
YWHAZ	Enzalutamide	−39.064
YWHAZ	BV02_9	−37.594
YWHAZ	MK2206	−32.715
YWHAZ	Corannulene	−23.241

Surprisingly, docetaxel outperformed BV02_9 in binding to the amphipathic groove of 14-3-3 proteins with ΔG values ranging from − 45.0 kcal/mol (YWHAB) to − 62.6 kcal/mol (YWHAZ) (Fig. 1e, Table 1). Consistent with expectations corannulene is a poor binder of 14-3-3 protein family members with ΔG ranging from − 18.0 kcal/mol (YWHAE) to − 29.7 kcal/mol (YWHAH). In summary, our docking results show that BV02 binds to a range of 14-3-3 protein family members.

### Perturbation of PCa cells with BV02: cell viability

Based on our docking data we established that BV02 and BV02_9 bind all 14-3-3 protein family members. We hypothesized that BV02 will affect cell viability similar to previous siRNA knock-down studies of YWHAZ carried out in a metastatic PCa model in which a 50% reduction in cell viability was observed [8]. We carried out the perturbation in cell culture using metastatic PCa models termed LNCaP (clone FGC) and PC-3. Unexpectedly, our results show no statistically significant change in cell viability of BV02 treated LNCaP or PC-3 cells compared to DMSO treated control (Additional file 1: Figure S3). Taken together, inhibition of 14-3-3 proteins using BV02 results in no statistically significant change in cell viability while knock-down of YWHAZ with siRNAs using PCa models results in a decrease in cell viability.

### Perturbation of PCa cells with BV02: proteome changes

To establish that BV02 does alter the proteome of perturbed PCa models, we quantified the proteome following perturbation of PCa models with BV02. The experiment was carried out in triplicate for each vehicle-treated (DMSO) and BV02 treated condition. Cells were exposed to BV02 for 24 h and then harvested, the proteome isolated and digested using sequence-specific serine protease trypsin. Following digest, peptides were purified and analyzed by LC-MS/MS. Mass spectrometry data were annotated using Andromeda search engine and label-free quantification carried out using the MaxQuant software. For subsequent analysis, only proteins and an adjusted *p*-value below 0.05 were considered regulated.

### Data integration with 14-3-3 interactome

**Fig. 2:**
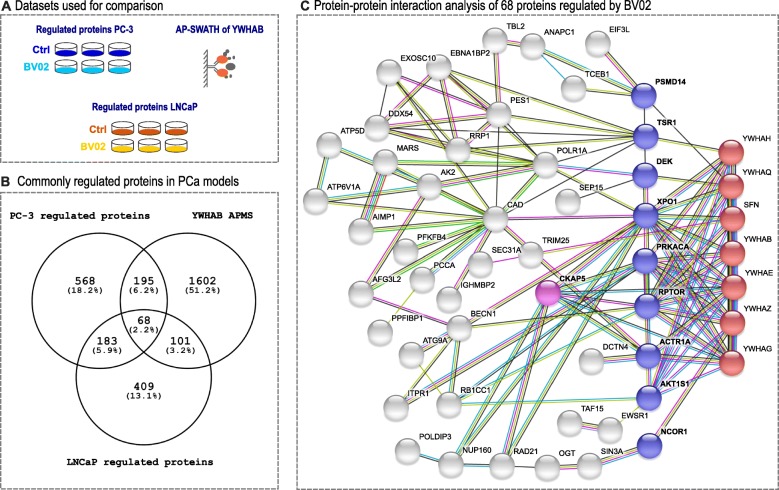
Data sets integration. **a** Three data sets were used for data integration: LNCaP clone FGC and PC-3 cells perturbed with BV02 and highly regulated proteins integrated with the YWHAB interactome. **b** There are 68 proteins shared between all three proteomic data sets. **c** These 68 proteins form a tightly connected protein-protein interaction network (STRING-DB v10.5). 14–3-3 proteins shown with red dots directly interact with ten proteins (in blue and pink), while all other regulated proteins do not directly interact with 14–3-3 proteins

BV02 binds to the amphipathic groove of 14-3-3 proteins and thus inhibits binding of other proteins via a consensus phosphorylated peptide motif. Our shotgun proteomics data detects many large changes in protein abundance as a consequence of BV02 treatment. To distinguish between direct interruption of protein-protein interactions due to BV02 and indirect or secondary effects we integrated our data set with an affinity purification data set in which a 14-3-3 protein was used as bait and interacting proteins identified using LC-MS/MS (14-3-3-APMS, Fig. 2a) [9]. 68 proteins were common between all three datasets (14-3-3-APMS, highly regulated proteins in LNCaP and PC-3 cells upon BV02 treatment) (Fig. 2b). Analyzing these 68 proteins for protein-protein interactions in STRING-DB v10.5 revealed a tight protein-protein network. Upon further inspection two layers of interactors were identified: there are eleven primary proteins interacting directly with 14-3-3 protein family members (labelled in blue/purple, Fig. 2c) while the remaining proteins are only indirectly connected to 14-3-3 protein family members (labelled in grey, Fig. 2c). Additional file 1: Table S2 lists all eleven proteins directly interacting with at least one 14-3-3 protein family member. Of interest are proteins involved in AKT / mTOR signalling, such as AKT substrate 1 (AKTS1), protein kinase C (PRKCA) and regulatory-associated protein of mTOR (RPTOR). Further, of these eleven proteins, only three are not involved in cell cycle progression: DEK, TSR1, and AKTS1. To find 14-3-3 proteins which directly bind to proteins involved in AKT / mTOR signal transduction and proteins involved in cell cycle progression gives additional support to the hypothesis that by targeting structural proteins multiple pathways might be inhibited. Of special note in this context is that cytoskeleton associated protein 5 (CKAP5, labelled in purple in Fig. 2c) has both direct protein-protein interactions between 14-3-3 protein family members and other proteins directly interacting with 14-3-3 proteins giving CKAP5 a unique position in this protein-protein network.

Although we could not establish any compelling evidence for the use of BV02 in our PCa models, other models might benefit from the drug. For example, BV02 (at the concentration used in our experiments) induced a 60% reduction in cell viability using chronic myeloid leukaemia [6].

## Conclusions

Structure-based drug design was used to assess binding specificity of inhibitor BV02 and related compounds for all seven human 14-3-3 proteins, revealing promiscuous binding. Surprisingly, docetaxel was identified as a strong binder to the amphipathic binding groove of 14-3-3 proteins, especially for YWHAZ. In vivo experiments on PCa models demonstrate that BV02 does modulate direct protein binders of 14-3-3 proteins. However, modulation of binding using BV02 and downregulation of 14-3-3 proteins using siRNAs have different effects in PCa models.

## Additional files

Additional file 1: Containing detailed description of materials and methods, modeling, supplementary tables and supplementary figures. (DOC 2230 kb)

## Abbreviations

AKTS1: AKT substrate 1
CKAP5: cytoskeleton associated protein 5
CRPC: castrate-resistant prostate cancer
ESCRT: Endosomal Sorting Complex Required For Transport
LC-MS/MS: Liquid chromatography coupled to mass spectrometer
mCRPC: Metastatic castrate-resistant prostate cancer
PCa: prostate cancer
PRKCA: protein kinase C
RPTOR: regulatory-associated protein of mTOR
