# Supplementary material for: Structure-based assessment and network analysis of targeting 14-3-3 proteins in prostate cancer
Source: Mol Cancer. 2018 Oct 31;17:156. doi: 10.1186/s12943-018-0905-y (PMC6208026; doi:10.1186/s12943-018-0905-y)
Supplement: Supplementary file 1 — Containing detailed description of materials and methods, modeling, supplementary tables and supplementary figures. (DOC 2230 kb) [file 12943_2018_905_MOESM1_ESM.doc]

**Structure-based Assessment and Network Analysis of Targeting 14-3-3 Proteins in Prostate Cancer**

**Alex Root ^1^, Azadeh Beizaei ^2^ and H. Alexander Ebhardt ^2^,***

^1^ Molecular Biology Program, Memorial Sloan Kettering Cancer Center, New York, NY, USA

^2^ Systems Biology Ireland, University College Dublin, Belfield, Dublin 4, Ireland

# Materials and Methods

**Cell Culture and Drug Perturbations**

LNCaP clone FGC (ATCC® CRL-1740™) and PC-3 (ATCC® CRL-1435™) are defined models available from American Type Culture Collection, Manassas, VA, U.S.A., kept below 20 passages and grown in 10 cm petri dishes in a 37 °C incubator with 5 % CO_2_ using RPMI-1640 or DMEM medium, respectively, supplemented with 10 % BSA, 2 mM L-glutamine, 100 units/mL Penicillin and 100 µg/mL Streptomycin. Monthly mycoplasma testing remained negative throughout the course of culturing. For perturbation experiments 2 million cells were seeded in a 10 cm dish, left to settle overnight and carried out in biological triplicate. BV02 was added to cell culture media to a final concentration of 5 μM. As vehicle treatment 5 μL DMSO were added to cell cultures. After 24 hours of treatment, dishes were placed on ice, media was aspirated, and cells were washed with 1xPBS (phosphate buffered saline) three times, scraped and pipetted into 1.5 ml tubes. Cells pellets were stored at -80 °C until further use.

**Quantification of Drug Response with Discovery Proteomics**

Cell pellets were lysed using urea buffer, alkylated, digested using sequence specific protease trypsin and peptides purified using HILIC (hydrophilic interaction chromatography). A step-by-step protocol is added as **Supplementary_Data_1**. Approximately 2 μg of purified peptides were injected per LC-MS/MS analysis using an Ultimate3000 nano-LC system coupled to a hybrid quadrupole-orbitrap mass spectrometer (Q Exactive, Thermo Fisher Scientific). Exact parameters are given in the Supplementary information. All .raw files, MaxQuant parameter file in xml format and output files are deposited under MassIVE ID MSV000081938 and are accessable through <ftp://massive.ucsd.edu/MSV000081938> . .

**Network Analysis of Drug Response**

Pathway enrichment was carried out using REACTOME. Perturbation data were mapped onto the STRING pathway database v10.5. All reaction types were retained. All proteins with log2 ratios greater than 1 or less than -1 were considered altered and retained. All 14-3-3 members were assumed to be altered and also retained.

**Structure-based drug design: details**

Structure-based drug design (SBDD) is now part of the early stages of drug discovery pipelines and is useful to screen compound libraries *in silico*, optimize lead hits, and guide researchers in understanding mechanisms of drug binding and action [1]. Docking uses scoring functions to assess relative binding affinities of small molecules to protein binding pockets using a molecular mechanics energy function with a distance-dependent dielectric model and calculation [2]. Docking can be performed using the Glide method which exhaustively searches position, orientation, and conformation spaces that ligands might inhabit, while remaining fast enough to screen large libraries [3]. Glide calculates a composite scoring function based on molecular mechanics and a distance-dependent dielectric model; ligand-binding pocket molecular mechanics interaction energy and ligand strain energy [3]. To overcome the limitations of using a single, rigid protein structure, Glide scales van der Waals radii of atoms in the protein and ligand to create additional space in the binding pocket [3]. While docking is useful to compare ligand binding affinities against a single protein, its results are not generally comparable among proteins [4]. To improve cross-comparability, techniques that estimate free energy of binding are used. The molecular mechanics generalized Born solvent accessibility method (MM-GBSA) is an intermediate between empirical scoring with docking methods and strict alchemical perturbation methods. The method estimates free energy of binding as the sum over bonded, electrostatic, and van der Waals interactions, plus polar and nonpolar contributions to solvation free energies, with the last term being temperature multiplied by entropy, which is estimated by a normal-mode analysis of vibrational frequencies [4].

**Preparation of Sequences, Structures and Ligands**

Sequences for all seven 14-3-3 family members were downloaded in FASTA format from UniProt KnowledgeBase with accession numbers given in **Supplementary Table 1** [5]. A multiple sequence alignment was generated using default parameters with Clustal Omega [6]. The multiple sequence alignment was visualized with Jalview version 2 [7]. Structures were imported from the Protein Data Bank, RSC PDB [8], into Maestro which is the centralized workspace of the Schrodinger Software Suite [9]. Protein structures given in **Supplementary Table 1** were prepared using the Protein Preparation Wizard [10]. For import and processing of protein structures the following settings were used: preprocess the structure with the following turned on: assign bond order; use CCD database, add hydrogens, create zero-order bonds to metals, delete waters beyond 5 Å from het groups, generate het states using Epik pH 7.2 +/- 0.0. Any missing side chains found were added. Refine the structure by optimizing the following H-bond assignment settings turned on: sample water orientations, use PROPKA pH 7.2. Remove waters with less than three H-bonds to non-waters. Delete dimers and ligands so that only a monomer structure remains. Chemical structures of ligands listed in **Supplementary Table 1** were exported from PubChem as structure data files (.SDF) and imported into Maestro. Ligands were prepared using the LigPrep application in the Schrodinger Software Suite [11]. The following settings were used: OPLS3 force field; ionization was set to generate possible confomers at target pH 7.2 +/- 0.0 using Epik, with desalt and generate tautomers. For computation of stereoisomers, chiralities were determined from 3D structure, with at most 32 per ligand.

**Docking Ligands and Estimating Free Energy of Binding**

Docking was performed using Glide (grid-based ligand docking with energetics) within the Schrodinger Software Suite [12]. Using the prepared protein structures and ligands in **Supplementary Table 1**, the first step in docking with Glide is grid generation, which specifies the active site region with the selection of site points that are spaced on a 2 Å grid. The active site where phosphopeptides bind on SFN are Lys-49, Arg-56, Arg-129, and Tyr-130 **Supplementary Figure 1** [13]. The receptor grid was generated by centroiding of the selected residues comprising the negatively charged portion of the binding pocket, with the ligand constrained to its diameter's midpoint within a bounding cube of 20 Å length, width, and height. No additional constraints were added. No hydroxyl or thiol groups in the protein were allowed to rotate. No volumes in the protein were excluded. For YWHAZ the active site residues were specified as Lys-49, Arg-56, Arg-127, and Tyr-128 according to work by Molzan and colleagues [14]. For the remaining five 14-3-3 proteins, the residues were specified using homology according to the multiple sequence alignment shown in **Supplementary Figure 2**: beta K51, R57, R129, Y130; gamma K50, R56, R132, Y133; epsilon K50, R56, R130, Y131; eta K50, R56, R132, Y133; tau K49, R55, R127, Y128s. The following settings were used for ligand docking. Scaling of van der Waals radii: scaling factor 0.80, partial charge cutoff 0.15. Conformer generation: energy window for ring sampling: 2.5 kcal/mol; canonicalize input conformation: turned on. Selection of initial poses: keep 5000 poses per ligand for the initial phase of docking; scoring window for keeping initial poses 100.0; keep best 800 poses per ligand for energy minimization. Energy minimization: distance-dependent dielectric constant: 2.0; maximum number of minimization steps: 100. Force field: OPLS3. Docking settings: XP (extra precision); ligand sampling: flexible; sample nitrogen inversions; sample ring conformations; bias sample of torsions for amides only with penalize nonplanar conformation; add Epik state penalties to docking score. No core, constraints or torsional constraints added. Filter to reject poses with Coulomb-vdW energy greater than 0.0 kcal/mol. Clustering: discard pose as duplicate if both RMS deviation is less than 0.5 Å and maximum atomic displacement is less than 1.3 Å; include hydroxyl/thiol hydrogens at 40 degree; strain correction: threshold for strain correction 4.00 kcal/mol; scaling factor for excess strain energy 0.25. Output settings: perform post-docking minimization; number of poses per ligand to include 10; threshold for rejecting minimized pose 0.50 kcal/mol. Write per-residue interaction scores for residues within 12 Å of receptor grid center. 2D visualization of residues responsible for binding affinity with drug ligands was performed with the Ligand Interaction Diagram feature of the Schrodinger Software Suite. Glide score summaries were provided in the Project Table feature of the Schrodinger Software Suite. Binding free energies were estimated for each ligand and 14-3-3 family member using the molecular mechanics, Generalized-Born solvation accessibility model (MM-GBSA). A custom implementation of the MM-GBSA method is provided by the Prime software suite within the Schrodinger Software Suite [15]. Protein-ligand complexes were taken from the Maestro pose viewer files with the following settings: solvation model VSGB; force field OPLS3; protein flexibility none; sampling method minimize.

In **Figure 1**, H-bonds are represented as solid purple lines. Pi-cation interactions are red. Distances and angles determine the interactions displayed are:

- For hydrogen bonds: maximum distance = 2.5 Å, minimum donor angle = 120.0°, minimum acceptor angle = 90.0°. These values are consistent with the default values used in Maestro.
- For salt bridges: Salt bridges are defined by oppositely-charged atoms that are within 5 Å and are not directly hydrogen-bonded.
- For pi-pi stacking: A pi-pi interaction is defined as an interaction between two aromatic rings in which either (a) the angle between the ring planes is less than 30° and the distance between the ring centroids is less than 4.4 Å (face-to-face), or (b) the angle between the ring planes is between 60° and 120° and the distance between the ring centroids is less than 5.5 Å (edge-to-face).
- Pi-cation interaction: The maximum distance between the cation center and the ring center is 6.6 Å and the angle between the ring plane and the line between the cation center and the ring center does not deviate from the perpendicular by more than 30°.
- For metal-coordination: The maximum distance between the metal and the ligating atom is 2.5 Å.
- The Ligand Interaction Diagram doesn't show hydrophobic interactions.

**Peptide generation and LC-MS/MS analysis**

Cell pellets were lysed using urea buffer, alkylated and digested using sequence specific protease trypsin. Peptide purification method included a HILIC (hydrophilic interaction chromatography) purification step in order to remove neutral lipids [16]. A step-by-step protocol is added as Supplementary files ([Step-by-step-cleanup-protocol.pdf](ftp://massive.ucsd.edu/MSV000081938/updates/2018-10-30_hebhardt_0db8346a/methods/Step-by-step-cleanup-protocol.pdf) deposited at <ftp://massive.ucsd.edu/MSV000081938> ).

Approximately 2 μg of purified peptides were injected per LC-MS/MS analysis using an Ultimate3000 nano-LC system coupled to a hybrid quadrupole-orbitrap mass spectrometer (Q Exactive, Thermo Fisher Scientific, Bremen, Germany). Peptides were separated by an increasing acetonitrile gradient from 2 % to 35 % on a C18 reverse phase chromatography column packed with 2.4 µm particle size, 300 Å pore size C18 material (Dr. Maisch GmbH, Ammerbuch-Entringen, Germany) to a length of 120 mm in a column with a 75 µm ID, using a flow rate of 250 nL/min. All data were acquired with the mass spectrometer operating in automatic data-dependent acquisition mode (DDA, shotgun). A full MS service scan at a resolution of 70,000, AGC target 3e6 and a range of *m/z* 350–1600 was followed by up to 12 subsequent MS/MS scan with a resolution of 17,500, AGC target 2e4, isolation window *m/z* 1.6 and a first fix mass of *m/z* 100. Dynamic exclusion was set to 40 sec. Raw instrument files are available in MassIVE ID <ftp://massive.ucsd.edu/MSV000081938> .

Protein identification from the MS/MS data was performed using the Andromeda search engine in MaxQuant (version 1.6.0.1; http://maxquant.org/) which matched data acquired by the mass spectrometer to the annotated canonical human [9606] proteome database obtained from UniProt KnowledgeBase (www.uniprot.org) along with common contamination sequences. The following search parameters were used: first search peptide tolerance of 20 ppm, second search peptide tolerance 4.5 ppm, fixed modification of cysteine carbamidomethylation, variable modifications of N-acetylation of protein and oxidation of methionine, use label free quantification analysis, match between runs, do not require MS/MS for quantification, and a maximum of two missed cleavage sites were allowed. False Discovery Rates (FDR) were set to 1 % for both peptides and proteins and the FDR was estimated following searches against a target-decoy (decoy = reverse of the database) database. LFQ intensities were calculated using the MaxLFQ algorithm [17] from razor and unique peptides with a minimum ratio count of two peptides across samples. Peptides with a minimum length of seven amino acids were considered for identification. Parameter file in xml format and output files of MaxQuant software are deposited under MassIVE ID <ftp://massive.ucsd.edu/MSV000081938> .

**Supplementary Table 1 Proteins and small molecules analyzed by SBDD, their sources and descriptions.**

| **Molecule** | **Database IDs** | **Source & Description** |
| --- | --- | --- |
| **YWHAB** | UniprotKB: P31946  PSC PBD: 2BQ0 | Structure of beta at 2.5 Å [18]. |
| **YWHAG** | Uniprot: P61981  PSC PBD: 3UZD | Structure bound to histone deacetylase 4 at 1.86 Å [19] |
| **YWHAE** | UniprotKB: P62258  PSC PBD: 2BR9 | Homodimer structure complexed with a peptide at 1.75 Å [18]. |
| **YWHAH** | UniprotKB: Q04917  PSC PBD: 2C63 | Homodimer structure complexed with a peptide to 2.15 Å [18]. |
| **SFN** | UniprotKB: P31947  PSC PBD: 1YWT | SFN in complex with a mode I-binding motif phosphopeptide at 2.4 Å [13]. |
| **YWHAQ** | UniprotKB: P27348  PSC PBD: 2BTP | Homodimer structure complexed with a peptide at 2.8 Å [18]. |
| **YWHAZ** | UniprotKB: P63104  PSC PBD: 4IHL | YWHAZ dimer bound to RAF and cotylenin A at 2.2 Å [14]. |
| **BV02** | PubChem: 1087336 | SFN inhibitor first reported nonpeptide inhibitor of 14-3-3 proteins. Possibly chemically unstable at room temperature and converts to BV02_9 [20]. |
| **BV02_9** | PubChem: 1087337 | SFN inhibitor that may represent the bioactive form of BV02 [20]. |
| **Docetaxel** | PubChem: 148124 | Microtubule depolymerization inhibitor used to treat mCRPC and is not expected to bind 14-3-3 proteins. |
| **Enzalutamide** | PubChem: 15951529 | Androgen receptor inhibitor that used to treat mCRPC and is not expected to bind 14-3-3 proteins. |
| **MK2206** | PubChem: 24964624 | Allosteric AKT1/2 inhibitor used in preclinical studies of PCa and is not expected to bind 14-3-3 proteins. |
| **Corannulene** | PubChem: 11831840 | A large organic compound with a 3D structure of a flat ring without any N, O, S atoms that is not expected to bind 14-3-3 proteins. |

**Supplementary Table 2:** Proteins directly interacting with 14-3-3 protein family members highly regulated by BV02 treatment in LNCaP and PC-3 cells.

| **Entry** | **Gene name** | **Protein names** | **Length** |
| --- | --- | --- | --- |
| P61163 | ACTR1A | Alpha-centractin (Centractin) | 376 |
| Q96B36 | AKT1S1 | Proline-rich AKT1 substrate 1 | 256 |
| Q14008 | CKAP5 | Cytoskeleton-associated protein 5 | 2032 |
| P35659 | DEK | Protein DEK | 375 |
| O75376 | NCOR1 | Nuclear receptor corepressor 1 | 2440 |
| P17612 | PRKACA | cAMP-dependent protein kinase catalytic subunit alpha | 351 |
| P17252 | PRKCA | Protein kinase C alpha type | 672 |
| O00487 | PSMD14 | 26S proteasome non-ATPase regulatory subunit 14 | 310 |
| Q8N122 | RPTOR | Regulatory-associated protein of mTOR | 1335 |
| Q2NL82 | TSR1 | Pre-rRNA-processing protein TSR1 homolog | 804 |
| O14980 | XPO1 | Exportin-1 | 1071 |

**
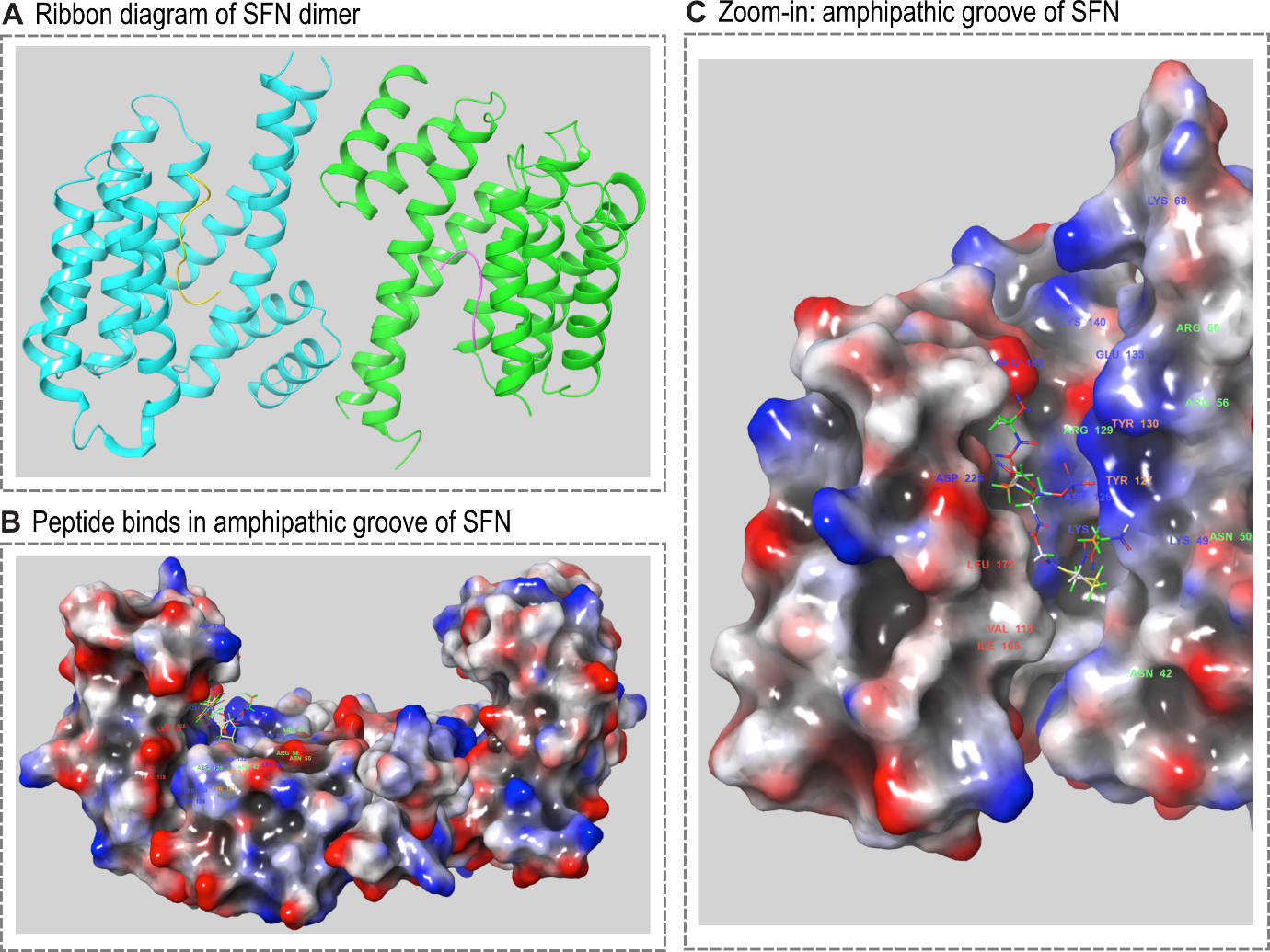
**

**Supplementary Figure 1. Phosphorylated peptide bound to SFN. A)** Ribbon diagram of SFN dimer (PDBID: 1YWT) consisting of two monomer with seven alpha helixes in complex with phosphorylated peptides which bind in amphipathic groove. **B)** Space-filling model colored by electrostatic potential showing the characteristic clamp or anvil structure of SNF. **C)** Detailed view of amphipathic groove occupied with phosphorylated peptide. Binding occurs in a cluster of basic residues shown in blue.

**
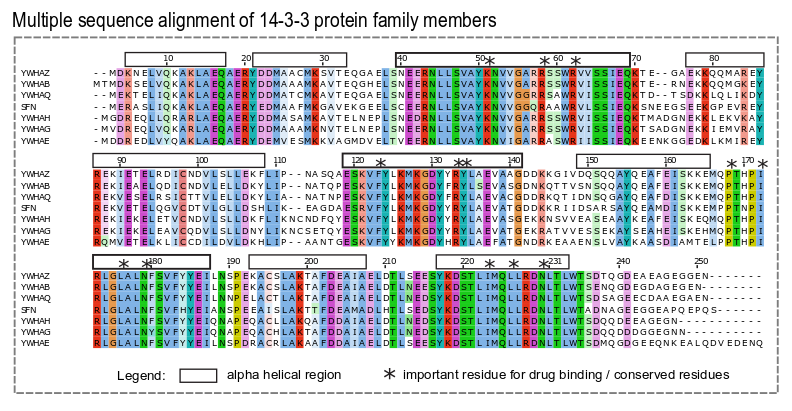
**

**Supplementary Figure 2. Multiple sequence alignment (MSA) of all seven 14-3-3 family members in human.** The MSA shows the high degree of conservation between the 14-3-3 protein family members. Colored residues are highly conserved between protein family members. ɑ helical regions are marked with square boxes while residues critical for drug binding are marked with an asterisk.


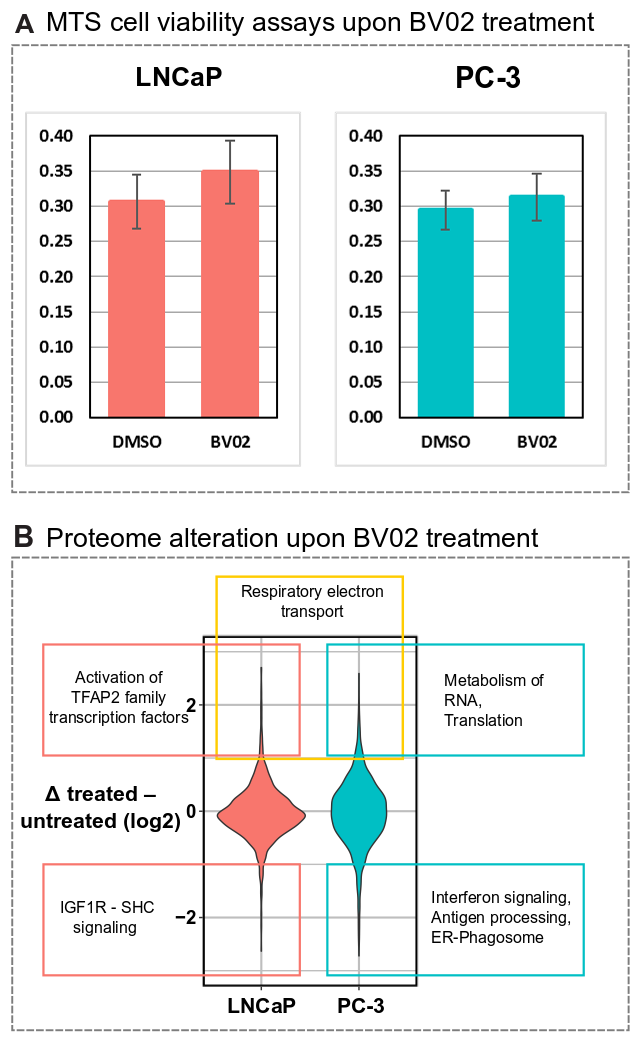


**Supplementary Figure 3**: **BV02 treatment of PCa cells**. **A)** MTS assay showing increased activity upon treatment of cells with BV02. **B)** Proteome analysis using high mass accuracy mass spectrometry confirms that in both cases up-regulated proteins are involved in energy metabolism (respiratory electron transport). However, down-regulated proteins differ: in LNCaP IGF1R-SHC signaling is effected while in PC-3 interferon signaling, antigen processing and ER-Phagosome pathway are regulated.

# References

1. Sledz P, Caflisch A: Protein structure-based drug design: from docking to molecular dynamics. Current opinion in structural biology 2017, 48:93-102.

2. C BR, Subramanian J, Sharma SD: Managing protein flexibility in docking and its applications. Drug Discov Today 2009, 14(7-8):394-400.

3. Friesner RA, Banks JL, Murphy RB, Halgren TA, Klicic JJ, Mainz DT, Repasky MP, Knoll EH, Shelley M, Perry JK *et al*: Glide: a new approach for rapid, accurate docking and scoring. 1. Method and assessment of docking accuracy. J Med Chem 2004, 47(7):1739-1749.

4. Genheden S, Ryde U: The MM/PBSA and MM/GBSA methods to estimate ligand-binding affinities. Expert Opin Drug Discov 2015, 10(5):449-461.

5. The UniProt C: UniProt: the universal protein knowledgebase. Nucleic Acids Res 2017, 45(D1):D158-D169.

6. Sievers F, Wilm A, Dineen D, Gibson TJ, Karplus K, Li W, Lopez R, McWilliam H, Remmert M, Soding J *et al*: Fast, scalable generation of high-quality protein multiple sequence alignments using Clustal Omega. Mol Syst Biol 2011, 7:539.

7. Waterhouse AM, Procter JB, Martin DM, Clamp M, Barton GJ: Jalview Version 2--a multiple sequence alignment editor and analysis workbench. Bioinformatics 2009, 25(9):1189-1191.

8. Berman HM, Westbrook J, Feng Z, Gilliland G, Bhat TN, Weissig H, Shindyalov IN, Bourne PE: The Protein Data Bank. Nucleic Acids Res 2000, 28(1):235-242.

9. Schrödinger: Schrödinger Release 2017-4: Maestro. In*.* New York, NY; 2017.

10. Sastry GM, Adzhigirey M, Day T, Annabhimoju R, Sherman W: Protein and ligand preparation: parameters, protocols, and influence on virtual screening enrichments. J Comput Aided Mol Des 2013, 27(3):221-234.

11. Schrödinger: Schrödinger Release 2017-4: LigPrep. In*.* New York, NY; 2017.

12. Fazi R, Tintori C, Brai A, Botta L, Selvaraj M, Garbelli A, Maga G, Botta M: Homology Model-Based Virtual Screening for the Identification of Human Helicase DDX3 Inhibitors. J Chem Inf Model 2015, 55(11):2443-2454.

13. Wilker EW, Grant RA, Artim SC, Yaffe MB: A structural basis for 14-3-3sigma functional specificity. J Biol Chem 2005, 280(19):18891-18898.

14. Molzan M, Kasper S, Roglin L, Skwarczynska M, Sassa T, Inoue T, Breitenbuecher F, Ohkanda J, Kato N, Schuler M *et al*: Stabilization of physical RAF/14-3-3 interaction by cotylenin A as treatment strategy for RAS mutant cancers. ACS chemical biology 2013, 8(9):1869-1875.

15. Jacobson MP, Pincus DL, Rapp CS, Day TJ, Honig B, Shaw DE, Friesner RA: A hierarchical approach to all-atom protein loop prediction. Proteins 2004, 55(2):351-367.

16. De Schrijver E, Brusselmans K, Heyns W, Verhoeven G, Swinnen JV: RNA interference-mediated silencing of the fatty acid synthase gene attenuates growth and induces morphological changes and apoptosis of LNCaP prostate cancer cells. Cancer research 2003, 63(13):3799-3804.

17. Cox J, Hein MY, Luber CA, Paron I, Nagaraj N, Mann M: Accurate proteome-wide label-free quantification by delayed normalization and maximal peptide ratio extraction, termed MaxLFQ. Mol Cell Proteomics 2014, 13(9):2513-2526.

18. Yang X, Lee WH, Sobott F, Papagrigoriou E, Robinson CV, Grossmann JG, Sundstrom M, Doyle DA, Elkins JM: Structural basis for protein-protein interactions in the 14-3-3 protein family. Proc Natl Acad Sci U S A 2006, 103(46):17237-17242.

19. Xu C, Jin J, Bian C, Lam R, Tian R, Weist R, You L, Nie J, Bochkarev A, Tempel W *et al*: Sequence-specific recognition of a PxLPxI/L motif by an ankyrin repeat tumbler lock. Sci Signal 2012, 5(226):ra39.

20. Valensin D, Cau Y, Calandro P, Vignaroli G, Dello Iacono L, Chiariello M, Mori M, Botta M: Molecular insights to the bioactive form of BV02, a reference inhibitor of 14-3-3sigma protein-protein interactions. Bioorg Med Chem Lett 2016, 26(3):894-898.
